# Magnitude of metabolic syndrome and its associated factors among patients with type 2 diabetes mellitus in Ayder Comprehensive Specialized Hospital, Tigray, Ethiopia: a cross sectional study

**DOI:** 10.1186/s13104-019-4609-1

**Published:** 2019-09-18

**Authors:** Gebreamlak Gebremedhn Gebremeskel, Kalayou Kidanu Berhe, Desta Siyoum Belay, Berihu Hailu Kidanu, Assefa Iyasu Negash, Kfle Tekulu Gebreslasse, Degena Bahrey Tadesse, Mulugeta Molla Birhanu

**Affiliations:** 1 Department of Nursing, College of Health Science, Aksum University, Aksum, Ethiopia; 2 Department of Adult Health Nursing, School of Nursing, College of Health Science, Mekelle University, Mekelle, Ethiopia

**Keywords:** Ethiopia, Associated factors, Magnitude, Metabolic syndrome

## Abstract

**Objective:**

The objective of this study was to assess magnitude of metabolic syndrome and its associated factors among type 2 diabetes mellitus patients in Ayder Comprehensive Specialized Hospital. A hospital based cross sectional study design was used. Binary logistic regression model was used.

**Result:**

A total of 419 respondents (208 males and 211 females) were enrolled; the mean age was 56.39 (SD 10.18), 51.1% of the respondents had metabolic syndrome according to international diabetes federation. Sex and age were statistically associated with metabolic syndrome with [AOR (95% CI) 1.93 (1.057, 3.533) and 1.04 (1.012, 1.072)] respectively. Regular physical exercise, overweight and obesity were statistically associated with metabolic syndrome with [AOR (95% CI) 1.84 (1.002, 3.362), 2.68 (1.518, 4.747) and 3.55 (1.254, 10.074)] respectively. To conclude, Magnitude of metabolic syndrome was high. The associated factors for metabolic syndrome are physical inactivity, inadequate intake of fruits, family history, overweight, and obesity.

## Introduction

Metabolic syndrome was defined by, WHO, NCEP ATP III and IDF etc. National cholesterol education program’s adult treatment panel III (NCEPATP III) defines metabolic syndrome as having three or more of the five risk factors: raised fasting plasma glucose, high blood pressure, abdominal obesity, low HDL, and/or raised plasma triglyceride [1]. The world health organization (WHO) defines metabolic syndrome as insulin resistance [impaired glucose tolerance (IGT) or diabetes] to be present for the diagnosis to be made. In addition to insulin resistance, at least two other components must also be present: obesity, hypertension, dyslipidemia and/or micro albuminuria [2].

Because of the study patients are type 2 DM, we select IDF criteria to define MetS. This is particularly evident for the risk of type 2 DM, which is apparent at much lower levels of obesity in Asians compared to Europeans. The IDF, having recognized the difficulties in identifying unified criteria for MetS that were applicable across all ethnicities, has proposed a new set of criteria with ethnic/racial specific cut-offs and IDF states that because of the limited data in Sub-Saharan Africa including Ethiopia, they should use European criteria [3].

According to the new international diabetes federation (IDF) definition, metabolic syndrome is defined as central obesity (defined by waist circumference) plus any two of the four risk factors: raised triglyceride, low high density lipoprotein (HDL) cholesterol, raised blood pressure and/or raised fasting plasma glucose level. Metabolic syndrome is a complex disorder which leads type 2 diabetes patients and cardiovascular disease (CVD) to be a twin global epidemic problem [4].

About 70–80% of diabetes mellitus (DM) [5] and 20–25% of adult population in the world is estimated to have metabolic syndrome and they are two times as likely to die from and three-fold as likely to have a heart attack or stroke as compared to without the syndrome. Besides, metabolic syndrome has five times increased risk of acquiring type 2 diabetes mellitus [6]. Consumption of calorie-dense foods, sedentary lifestyle, and tobacco consumption are considered as potential risk factors for MetS [7]. In Ethiopia, the prevalence of metabolic syndrome among type 2 diabetes mellitus according to NCEP on (ATP) III was 45.9% [8].

There is limited data on metabolic syndrome in Ethiopia and in the horn of Africa at large. Therefore, this study is helpful to the development of life style modification, control and management of the metabolic syndrome. Finally, this will bring non-communicable disease in general and metabolic syndrome in particular into the clinical area, and research agenda.

## Main text

### Methods and patients

#### Study setting and period

A hospital based cross section study design was conducted in Ayder Comprehensive Specialized Hospital (ACSH) from February to June 2018. ACSH is found in Mekelle the capital city of Tigray region northern of Ethiopia. Mekelle is located around 780 km north of the Ethiopian capital city Addis Ababa. The study participants were previously diagnosed/known type 2 DM patients. DM clinic is served for about 500 type 2 DM patients per month that is estimated from the average of the last 6 months report.

Study design: A hospital based cross section study design.

Source patients: All type 2 diabetes patients attending DM clinic at ACSH.

Study patients: All volunteer type 2 DM patients attending DM clinic and available at ACSH during the data collection period.

Study subjects: All sampled subjects (419) participated in this study.

##### Inclusion criteria

- All type 2 diabetes mellitus patients.

##### Exclusion criteria

- Critically ill patient.
- Pregnant mother (difficult to measure BMI and waist circumference).

#### Sample size determination

The sample size was calculated by using single proportion formula, ${\text{n}} = \frac{{\left[ {{\text{Z }}\frac{\alpha }{2}} \right]^{2} {\text{p}}\left( {1 - {\text{p}}} \right)}}{{d^{2} }}$ at 95% confidence level, where, ${\text{Z}}\frac{\alpha }{2} = {\text{ standard normal deviation }}\left( { 1. 9 6} \right)$, d = 5% of marginal error, and p = prevalence 45.9% taken from a study conducted in Ethiopia [8].

$${\text{n }} = \frac{{\left( {1.96} \right)^{2} \times 0.459\left( {0.541} \right)}}{{\left( {0.05} \right)^{2} }},{\text{ n}} = { 382}$$

Considering 10% non-response rate, because of some respondents may withdraw in between the interview, the total estimated study subjects were 419 and selected by systematic random sampling technique with kth interval of 2 and every second (2) was participated by random selection from 1 to 2(kth).

#### Data collection procedure

Data collection was carried out from February to March 2018. To collect data a standardized, interviewer administered questionnaire and document review checklist of physical measurements, residence (urban or rural) for the last 6 month, co-morbid disease like hypertension, CHF, stroke were used and contains three parts. Thus are socio-demographic and medical history, the life style and related information.

#### Measurements and tools

Anthropometric measurements: Anthropometric measurements of weight and height was measured using Seca weighing scale and stadiometer respectively and participants were wearing light clothing (single and thin) and without shoes to the nearest Kg and CM respectively. A simple flexible steel metric tape calibrated in meters was used for measuring waist circumference. Waist circumference was measured midway between the iliac crest and the lower rib margin in the horizontal plane while the participant is standing to the nearest 0.5 cm.

##### Blood pressure

Two blood pressure measurements taken after 5 min apart were determined for each participant using standard adult digital blood pressure apparatus with the correct size arm cuff. Participants was measured after 5 min of rest in sitting position, arm should be rest on table at heart level, back supported, on the same arm and legs rest on ground (no crossed). And the average readings of the two measurements was recorded in questionnaire [9].

#### Data quality assurance

Questionnaire was prepared in English and translated into local language Tigrigna by expert in language. Pretest was done on 5% of the subjects in Mekelle hospital 2 weeks before the actual data collection. Because Mekelle hospital is near to ACSH and have similar socio-demographic characteristics with patients in ACSH. Two BSC nurses and one supervisor were recruited for data collection and training was given. The collected data was checked by supervisor and principal investigator daily.

#### Data processing and analysis

After the data collection, the data was entered into Epi-info version 7 and exported to SPSS version 23 statistical program. Descriptive characteristics were presented in text, frequency percentage tables, and graphs. Binary logistic regression model was used to see the association between the outcome variable with each independent variables. Then those variables with P-value < 0.25 at bivariate analysis was included in the multivariable analysis. Finally P-value < 0.05 is describe as a statistically associated with 95% confidence level. Model fitness was checked by using Hosmer and Lemeshow goodness fit model.

#### Operational definition

##### Metabolic syndrome

As per the definition of international diabetes federation it is defined as having central obesity (defined by waist circumference ≥ 94 cm for male and ≥ 80 cm for female) plus any two of the following four factors: raised triglycerides, reduced HDL cholesterol, raised blood pressure, and/or raised fasting plasma glucose.

##### Central obesity

defined by waist circumference ≥ 94 cm for male and ≥ 80 cm for female putting to the nearest centimeter.

##### Raised triglycerides

≥ 150 mg/dL (1.7 mmol/L) or specific treatment for this lipid abnormality.

##### Reduced HDL cholesterol

< 40 mg/dL (1.03 mmol/L) in male < 50 mg/dL (1.29 mmol/L) in female or specific treatment for this lipid abnormality.

##### Raised blood pressure

Is a systolic BP ≥ 130 mm Hg or diastolic BP ≥ 85 mm Hg, or any patient on treatment of previously diagnosed hypertension.

##### Reduce salt intake

About 1 tea spoon of table salt per meal.

### Result

#### Socio-demographic characteristics

A total of 419 type 2 DM patients were enrolled in this study. Binary logistic regression was used. Of these respondents 211 (50.4%) were females. The mean age of the respondents was 56.39 SD ± 10.18 with minimum of 35 and maximum of 90 years old. Of the total respondents 79.7% were married, 83.1% were orthodox Christian followers (Table 1).

**Table 1:** Frequency distribution of socio-demographic characteristics of respondents among type 2 diabetes mellitus patients in ACSH, Tigray, Ethiopia (n = 419)

Variable	Category	No.	Percent
Sex	Female	211	50.4
	Male	208	49.6
Ethnicity of participants	Tigray	395	94.3
	Amara	24	5.7
Marital status	Single	19	4.5
	Married	334	79.7
	Divorced	24	5.7
	Widowed	42	10
Religion	Orthodox	348	83.1
	Muslim	65	15.5
	Protestant	6	1.4
Occupation of participant	Farmer	50	11.9
	Merchant	110	26.3
	G. Employed	115	27.4
	Daily laborer	25	6
	House wife	78	18.6
	P. Employed	41	9.8
Educational status	Unable to read and write	131	31.3
	Primary school	118	28.2
	High school	69	16.5
	College and above	101	24.1
Place of residence	Urban	361	86.2
	Rural	58	13.8

N.B: G. employed: government employed, P. employed: private employed

#### Metabolic syndrome and its component

**Fig. 1:**
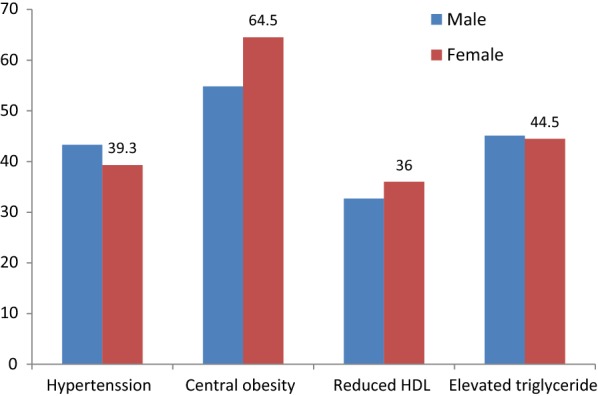
Frequency of component of MetS in relation to sex of respondents among type 2 patients with DM in ACSH, Tigray, Ethiopia, 2019 (n = 419)

According to IDF criteria 51.1% of the respondents had metabolic syndrome, with higher prevalence in females when compared to males (57.5% and 42.5% respectively). Central obesity (59.7%) was highly prevalent component of metabolic syndrome followed by elevated triglyceride (45.1%) and then hypertension and low HDL-c 41.3% and 34.4% respectively. Females were also having higher proportion of central obesity and reduced HDL than males (Fig. 1).

#### Factors associated to metabolic syndrome

In the multivariable analysis, age [AOR (95% CI) 1.04 (1.01, 1.07)], sex [AOR (95% CI) 1.94 (1.06, 3.54)], eating fruits once, twice, and four and above times in a typical week [AOR (95% CI) 0.41 (0.21, 0.78), 0.35 (0.16, 0.76), and 0.24 (0.07, 0.75)], regular physical exercise [AOR 1.83 (1.05, 3.36)], family history of DM [AOR (95% CI) 0.54 (0.29, 0.99)], chronic disease co-morbid [AOR 0.42 (0.25, 0.70)] Overweight [AOR (95% CI) 2.66 (1.50, 4.69)] and obesity [AOR 3.50 (1.23, 9.91)] shows statistical association with metabolic syndrome (Table 2).

**Table 2:** Multivariable analysis of the independent variables with metabolic syndrome among patients with type 2 diabetes mellitus in ACSH, Mekelle, Tigray, Ethiopia (n = 419)

Variables	Metabolic syndrome		Crude OR		Adjusted OR	
	No	Yes	P-value	95% CI	P-value	95% CI
Sex						
Male	117	91		1		1
Female	88	123	0.003	1.79 (1.22, 2.64)	*0.031*	1.94(1.06, 3.54)*
Age			0.00	1.05 (1.03, 1.08)	*0.005*	1.04 (1.01, 1.07)*
Ethnicity of participant						
Tigray (Ref)	187	208		1		1
Amara	18	6	0.012	0.3 (0.11, 0.77)	0.1357	0.40 (0.11, 1.42)
Marital status			0.068		0.549	
Single	14	5		1		1
Married	164	170	0.045	2.9 (1.02, 8.24)	0.287	2.01 (0.55, 7.32)
Divorced	12	12	0.12	2.8 (0.76, 10.24)	0.509	1.73 (0.34, 8.79)
Widowed	15	17	0.008	5.04 (1.52, 16.7)	0.157	2.97 (0.65, 13.50)
Occupation			0.002		0.322	
Farmer	37	13		1		1
Merchant	52	58	0.02	3.17 (1.52, 6.62)	0.062	3.36 (0.94, 12.01)
Government employee	57	58	0.04	2.89 (1.39, 6.01)	0.039	3.88 (1.07, 14.06)
Daily laborer	14	11	0.119	2.24 (0.82, 6.15)	0.294	2.25 (0.49, 10.24)
House wife	33	45	0.001	3.88 (1.78, 8.43)	0.073	3.3 (0.89, 12.22)
Private employee	12	29	0.00	6.87 (2.73, 17.31)	0.027	4.78 (1.19, 19.11)
Residence of participant						
Urban	163	198		1		1
Rural	42	16	0.00	0.31 (0.17, 0.57)	0.817	1.14 (0.37, 3.45)
Physical exercise						
Yes	60	34		1		1
No	145	180	0.001	2.19 (1.36, 3.52)	*0.048*	1.83 (1.05, 3.36)*
Eat fruits in a typical week			0.221		*0.027*	
None per week	54	78		1		1
One times a week	83	77	0.062	0.64 (0.4, 1.02)	*0.007*	0.41 (0.21, 0.78)*
Twice a week	42	36	0.07	0.59 (0.34, 1.04)	*0.008*	0.35 (0.16, 0.76)*
Three times a week	11	7	0.111	0.44 (0.16, 1.20)	0.088	0.31(0.08, 1.18)
Four and above a week	15	16	0.449	0.74 (0.36, 1.62)	*0.014*	0.24 (0.07, 0.75)*
Eat vegetablesin a typical week			0.028		0.068	
None per week	17	22		1		1
One times a week	37	21	0.051	0.43 (0.19, 1.05)	0.122	0.42 (0.14, 1.25)
Twice a week	69	58	0.242	0.65 (0.31, 1.33)	0.345	0.62 (0.23, 1.66)
Three times a week	28	43	0.672	1.18 (0.53, 2.62)	0.650	1.27 (0.44, 3.65)
Four and above a week	54	70	0.996	1.02 (0.48, 2.07)	0.718	1.21 (0.43, 3.40)
Any alcoholic drinks						
Yes	31	23		1		1
No	174	191	0.183	1.48 (0.83, 2.63)	0.611	1.23 (0.54, 2.78)
Ever smoke any tobacco product ever						
Yes	2	6		1		1
No	203	208	0.191	0.34 (0.68, 1.72)	0.092	0.17 (0.02, 1.33)
Low salt intake						
Yes	148	174		1		1
No	57	40	0.028	0.59 (0.37, 0.94)	0.344	0.074 (0.41, 1.36)
Body mass index			0.000		0.001	
Normal weight	147	108		1		1
Underweight	15	1	0.021	0.91 (0.01, 0.69)	0.089	0.14 (0.01, 1.34)
Overweight	37	76	0.000	2.79 (1.76, 4.45)	*0.001*	2.66 (1.50, 4.69)*
Obese	6	29	0.000	6.58 (2.64, 16.4)	*0.018*	3.50 (1.23, 9.91)*
Duration of DM			0.000		0.038	
< 5 years duration	134	84		1		1
6–10 years duration	46	63	0.001	2.18 (1.37, 3.48)	0.334	1.35 (0.73, 2.50)
> 10 years duration	25	67	0.000	4.27 (2.5, 7.29)	*0.010*	2.49 (1.23, 5.01)*
Treatment			0.012		0.073	
Metformin	70	57		1		1
Glibenclamide	13	4	0.104	0.38 (0.12, 1.22)	0.156	0.35 (0.08, 1.47)
Metformin plus glibenclamide	67	70	0.313	1.28 (0.79, 2.08)	0.604	0.84 (0.45, 1.58)
Insulin plus oral hypoglycemic agents	55	83	0.013	1.85 (1.14, 3.02)	0.148	1.62 (0.84, 3.12)
Family hx of DM						
Yes	33	74		1		1
No	172	140	0.000	0.36 (0.228, 0.58)	*0.047*	0.54 (0.29, 0.99)*
Chronic disease						
Yes	70	123		1		1
No	135	91	0.000	0.38 (0.26, 0.57)	*0.001*	0.42 (0.25, 0.70)*

N.B; * states significant association, and 1; reference category

### Discussion

This study was aimed to assess magnitude of metabolic syndrome and its associated factors among type 2 DM patients who have follow up in ACSH.

The magnitude of metabolic syndrome among type 2 DM patients in this study was 51.1%; which lies within a range of 12–86% from a review study done in sub Saharan Africa [10]. But, this study is higher than from a study conducted in Ethiopia (45.9%), in 10 European countries (24%) and UK (32%) [8, 11]. This difference is due to difference in study setting, sample size, and the criteria used to define metabolic syndrome. The magnitude of MetS in this study is lower than from studies done in Gahanna (68.6%), Nigeria (68.7%) and Iran (64.9%) [12–14].This variation could be due to difference in socio-cultural, study setting, study design and life style.

This study reveals that, age was significantly associated with metabolic syndrome, and this is in line with a study conducted in Iran [12]. This association might be due to older age participants were physically inactive and adoption of unhealthy life styles. Sex was another variable that shows statistical association with metabolic syndrome. This is consistent with studies in Hawassa Ethiopia, Addis Ababa Ethiopia, Nigeria, and Iran [8, 12, 13, 15].

Respondents who eat fruit twice, four and above times in a typical week were 65% and 76% protected from metabolic syndrome respectively as compared to those who never eat fruit in a typical week. This is supported by EPHA (Ethiopia) and in line with study conducted in brazil [16, 17].

Respondents who did not do regular physical exercise were 1.83 odds to have MetS as compared to those who did regular physical exercise. This is supported by EPHA report (Ethiopia) and in line with studies done in US, Canada, and Sub-Saharan Africa [16, 18–20].

In addition, the odds of MetS were 2.66 and 3.5 times higher among overweight and obesity compared to those normal weigh respondents respectively among type 2 DM patients. This is in line with studies done in Ethiopia, Gahanna, Iran and Nigeria [8, 12, 13, 21]. But lower in AOR from a study in Ethiopia. This may be due to sample size and difference in reference category.

### Conclusion and recommendation

#### Conclusion

The magnitude of metabolic syndrome among type 2 diabetes mellitus is 51.5%. The associated factors for metabolic syndrome are physical inactivity, advanced age, inadequate intake of fruits, family history, overweight and obesity.

#### Recommendations

##### To Ayder Comprehensive Specialized Hospital

- Regular screening of type 2 DM patients in ACSH for components of MetS is vital in order to avert/limit the risks before developing cardiovascular related morbidity and mortality.

##### To type 2 DM in ACSH

- Should do regular physical exercise as recommended.
- Should reduce and manage their weight if they are told to do so by health care professionals.

##### To further study

- Further cohort or interventional studies should be done to address other predictors of MetS.

## Limitation of the study

- The cross sectional nature of the study; temporal relationship between exposure and disease cannot be clearly determined or not powerful.
- The study was conducted only in a single public hospital.

## Data Availability

I the undersigned, declare that this is our original work and has not been submitted and considered for publish in any journal and all sources of materials and data used for this research have been secured and acknowledged. Data and materials were not shared publicly for confidentiality purpose.

## Abbreviations

ACSH: Ayder Comprehensive Specialized Hospital
DM: diabetes mellitus
EPHA: Ethiopian Public Health Association
HDL: high density lipoprotein
IDF: International Diabetes Federation
MetS: metabolic syndrome
NCEP/ATP: National Cholesterol Education Adult Treatment Panel
US: United States
